# Fine Comminution of Pine Bark: How Does Mechanical Loading Influence Particles Properties and Milling Efficiency?

**DOI:** 10.3390/bioengineering6040102

**Published:** 2019-11-06

**Authors:** Karine Rajaonarivony, Xavier Rouau, Komlanvi Lampoh, Jean-Yves Delenne, Claire Mayer-Laigle

**Affiliations:** IATE, Univ Montpellier, CIRAD, INRA, Montpellier Supagro, 34060 Montpellier, France; karine.rajaonarivony@supagro.fr (K.R.); xavier.rouau@inra.fr (X.R.); komlanvi.lampoh@inra.fr (K.L.); jean-yves.delenne@inra.fr (J.-Y.D.)

**Keywords:** milling efficiency, mechanical loading, lignocellulosic biomass, powder agglomeration

## Abstract

The use of lignocellulosic plant biomass as an alternative to fossil feedstocks for chemistry, energy and materials often involves an intense dry comminution step, for which the energy consumed can vary significantly according to the process parameters, the particle size targeted, and the properties of the biomass. Here we studied the fine milling of maritime pine bark in an impact-mill configuration and in an attrition-mill configuration. The properties of the resulting powders (particle size distribution, particle shape, specific surface area, agglomeration level) obtained in each configuration were compared in relation to process energy consumption. Results evidenced that the agglomeration phenomena drive milling efficiency and limit the possibilities for reaching ultrafine particles. Interestingly, impact loading proved more effective at breaking down coarse particles but tended to generate high agglomeration levels, whereas attrition milling led to less agglomeration and thus to finer particles.

## 1. Introduction

Comminution is achieved by applying repeated loading constraints to a raw material in order to exceed the failure stress at particle scale. The grinder or mill transmits the stresses down to the particles through different mechanical loadings, such as compression, impact, shearing, and abrasion/attrition [1]. These loading types have a strong influence on the characteristics of the ground powders and on the energy consumption of the process [2]. Lignocellulosic biomass involves strong anisotropy, a complex microstructure, and non-linear mechanical behavior. Efficient fragmentation of these materials implies successive milling steps adapted to the histological organization of the biomass in order to shift particle sizes from centimeter to micrometer ranges [3]. Furthermore, recent research has highlighted that high-level applications in energy, chemistry and materials fields demand ever finer biomass powders with target median sizes below 50 µm [4,5]. This extreme pulverization requires huge energy input and more advanced milling technologies that are still only nascent science. Small-scale experiments and a few industrial pilots [1] have highlighted two types of process technologies: opposed jet milling and media milling. In a jet mill, particles are severely collided together in pressurized air jets, where milling process efficiency is essentially governed by the air pressure. Particles obtained from jet milling are generally more elongated than those obtained from media milling [4]. Jet milling is relatively complex to handle and requires fairly high investment and operation costs, which limit its potential for use in industry. In media-milling processes, plant biomass particles are blended with the milling media (spherical or cylindrical or specially-shaped balls or beads) in a rolled chamber, a vibrated moving chamber or a stationary chamber equipped with a rotary agitator [6]. Biomass particles are impacted and worn down by the milling media. The process parameters (rotation speed, mass of milling media, filling ratio, and more) and the technology employed modulate the proportion of the different mechanical loadings involved in the comminution (i.e., compression, impact, shearing, and abrasion/attrition). Media mills generally require less power input than rotor or jet mills and involve lower investment per processed mass unit. For these reasons, media milling makes a good technological option for industrial-scale ultrafine comminution of lignocellulosic biomass presenting relatively low added value (for energy or materials applications).

However, a major process limitation often observed in media mills is the agglomeration phenomenon, which regularly occurs in ultrafine milling set-ups [7,8]. This is because a dramatic reduction in particle size diminishes the ratio between the force of gravity and inter-particle forces (van der Waals, capillary, electrostatic forces), thereby amplifying attraction and cohesion [9,10,11,12]. Lignocellulosic particles are particularly sensitive to these forces due to their low density, complex surface chemistry, and sensitivity to humidity. Fine-milling processes can also lead to in-depth chemical transformations, such as the breakdown of various bonds—including covalent bonds—inside the material, which generates free radicals that can persist for varying periods of time depending on ambient conditions and on the chemistry of the materials in the mill. Radical reactions strengthen the intensity of the electrostatic and van der Waals forces [13,14] and lead to stronger agglomeration [15,16]. This phenomenon, which can limit size reduction and even create larger particles through a clustering effect, has been reported and studied for inorganic nanoparticles, but it also occurs in micrometric lignocellulosic particles as their density is lower [14,15]. Mucsi [17] showed a size limit of 1 μm is reached for milling of quartz particles in a stirred media mill, and Karinkata [18] showed a size limit of 20 µm for wood sawdust particles in a vibratory ball mill.

As a result, the size of the ground particles and the energy cost of the milling operation are difficult to predict with traditional grinding laws [19,20]. Furthermore, agglomeration phenomena caused by clustering the smallest particles could also modify the flowability and end-use properties of the ground powders [21,22]. 

As the applications involving ultrafine plant powders are just emerging, the process of fine milling plant materials is still under-studied. However, reducing the production costs and optimizing the grade-standard quality of high-tech plant powders hinges on gaining a far better understanding of the comminution and agglomeration mechanisms occurring during the milling process. In-depth study of processes occurring in an operating media mill can advantageously be realized at small scale in a laboratory vibrating mixer mill. This kind of equipment makes it possible to use different types of grinding media with different sizes and weights and, therefore, to experiment various configurations that simply mimic the phenomena taking place in larger-scale mills. Here we investigated the fine milling of maritime pine bark in an impact-mill configuration and in an attrition-mill configuration. The properties of the resulting powders (particle size distribution, particle shape, specific surface area, agglomeration level) obtained in each configuration were compared in relation to process energy consumption.

## 2. Materials and Methods

### 2.1. Input Raw Materials

The lignocellulosic biomass used in this study is a commercial bark of maritime pine (BMP, *Pinus pinaster*) with pieces calibrated at between 10 and 25 mm intended for mulching applications. The bark was purchased in a local store (Botanic, Montpellier, France). BMP was first coarsely ground in a Retsch SM300 cutting mill (Retsch, France) equipped with a 2-mm sieving grid, operated at a rotary speed of 3000 rpm. After this first milling step, the BMP was over-dried at 105 °C to reach a final moisture content of 1.5%. The median size, d50, measured by laser granulometry and the apparent tapped density of this initial powder were 410 µm and 294 kg/m³, respectively. 

### 2.2. Milling Protocol

**Figure 1:**
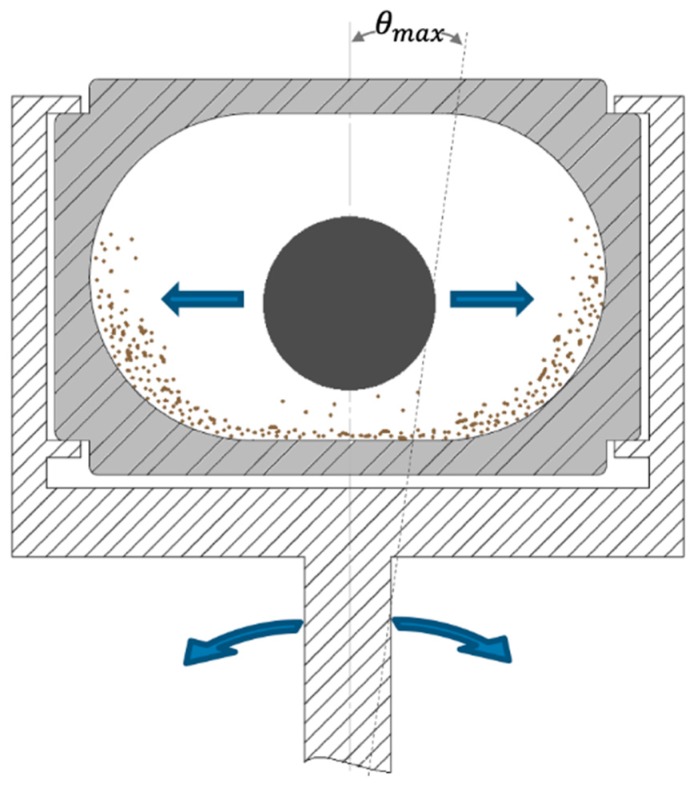
Scheme of the grinding jar in its holder on a single-ball mill. Arrows show ball movement and radial rotation of the holder.

The Retsch MM400 mill was chosen to compare fine milling of BMP under different mechanical stresses. The Retsch MM400 is a vibratory ball mill equipped with two 50-mL stainless-steel milling jars. Each milling jar is fixed on an arm that undergoes radial vibration in the horizontal plane, as illustrated in Figure 1.

The linear amplitude of the jar, measured using a contactless position sensor (MX1C-AK1 laser displacement sensor, Idec, Japan), was 7.2 mm, which corresponds to an angular amplitude of $\theta_{max}≃2.1^{\prime }$. The oscillation frequency can be varied from 0 to 30 Hz. In the jar, the shaking motion of one ball or several beads impacts the input material to be milled. Each jar was filled with 1.8 g (6.1 mL) of the input raw powder. Total media-milling weight was 63 g, either as a single 25 mm-diameter stainless steel ball (single-ball configuration) or as 73 6 mm-diameter stainless steel beads (multi-bead configuration). The filling ratio of the jars, taking into account milling media plus raw material, was 29% in single-ball configuration and 36% in multi-bead configuration. These filling ratios fit with those traditionally used for ball mills and with the device manufacturer’s recommendations. All milling experiments were performed at a 20 Hz frequency. The time–course evolution of particle size is rapid over the first few milling periods, but then advances more slowly. Thus, to describe the milling kinetics, based on preliminary trials, we opted for sampling at the following series of milling times: 0.5, 1, 2, 5, 20, 60, 100, 140 and 200 min. The milling experiments were duplicated and carried out at ambient temperature. The energy consumption of milling was recorded using a PX 120 power meter (Metrix, France) connected to WattCom software (Metrix, France). The time–course evolution of external jar temperature was also measured using an Optris laser probe (Optris, Germany) connected to AMR WinControl software (Ahlborn, Germany).

### 2.3. Discrete Element Simulations of Ball and Bead Trajectories

To clarify the different comminution mechanisms (impact, friction) operating in the two media configurations (single-ball, multi-bead), we investigated the dynamics of the jar/media system using discrete element method (DEM) simulations [23,24,25,26]. 

The model considers the forces due to gravity, Hertzian contact and Coulomb friction [27] between rigid bodies (particles to mill, balls and jar). Although the model is capable of accounting for a powder load, here, as a first approximation and for sake of computational efficiency, only the milling media (ball, beads) were considered. It is assumed that the additional dissipation due to the powder bed can be quantified through a damping coefficient which is only active during collisions and contacts.

The simulation iterates on two main steps until the simulation ends. First, corrected accelerations are computed from a force balance on each rigid body and using Newton’s second law of motion. Then, new positions are obtained thanks to the velocity Verlet integration scheme [28] and all forces are updated using contact laws. Jar motion is controlled in angular rotation (θ) using the following equation:

(1) $$\theta \left(t\right)=\theta_{max}\sin\left(2\pi ft\right),$$

where $\theta_{max}$ is the experimentally-determined stroke of the jar (see Section 2.2), and the frequency f is held at a constant 20 Hz in the simulations.

This study focuses on energy dissipation, as it is related to the comminution efficiency of the material.

### 2.4. Particle Size Distribution and Specific Surface Area

The particle size distribution (PSD) of the powder dispersed in ethanol (96% *v*/*v*) was measured by a Mastersizer 2000 laser diffraction granulometer (LG) (Malvern, UK) equipped with the Hydro 2000S system. The refractive index of wood sawdust, 1.53, was used to proceed with the data. All measurements were carried out in duplicate. Median particle size (d50) and specific surface area (SSA) were also extracted from the PSD. The SSA (in m^2^·kg^−1^), which corresponds to the total surface developed by the powder, was determined according to the following equation:

(2) $$SSA=\frac{1}{\rho }\sum_{i}\frac{3\alpha_{i}}{R_{i}},$$

where *ρ* is true density of the powder, *i* is index grading class (between 0.02 and 2000 μm), $\alpha_{i}$ is volume fraction and R_i_ is average radius of particles in the *i*th class. 

SSA was also determined using the Brunauer–Emmett–Teller (BET) method [29] based on nitrogen adsorption onto the particle surfaces. Samples of 1.0–1.5 g of powder were first degassed during 48 h at 50 °C using a Vacprep 061 degasser (Micrometrics, USA) to remove moisture and other adsorbed molecules, then analyzed using an ASAP 2460 analyzer (Micrometrics, USA). All BET SSA measurements, denoted SSA_BET_ in the following, were carried out in duplicate. 

### 2.5. Powder Agglomeration

In this work, we quantified powder agglomeration as the difference between the SSA of a powder as-is, measured by laser diffraction (SSA_agglo_, agglomerated SSA), and the SSA of the same powder de-agglomerated using either the Mastersizer Hydro 2000S’s ultrasound de-agglomerating system (SSAD-_LG_, granulometer system de-agglomerated SSA) or a more powerful external ultrasonic probe (SSAD-_EP_, external probe de-agglomerated SSA). The two corresponding protocols are described below.

*Powder de-agglomeration protocol using the Mastersizer ultrasonic system*: 3 minutes of sonication using the granulometer ultrasound probe at 100% of its power (75 W) was applied to a suspension of approximatively 0.1 g of powder in 200 mL of ethanol. The suspension was then stirred at 3000 rpm for 5 minutes to remove any bubbles prior to measurement of SSA D-_LG_.

*Powder de-agglomeration protocol using the external ultrasound probe*: a suspension of 0.1 g of powder in 200 mL of ethanol was stirred with a magnetic agitator then sonicated using a ¼” Qsonic Q700 ultrasound microtip probe (Qsonica, USA) at 130 W maximum power. Sonication time (5 min) and power applied (130 W) to the powder suspension were determined based on preliminary trials and correspond to the time that gives the best de-agglomeration while limiting excessive heating. Then, the median particle sizes and SSA were determined using laser granulometry after 30 s of sonication using the granulometer ultrasound probe at 100% of its power (75 W). Note that the ultrasound energy delivered in the external probe protocol (130 W, 5min + 75 W, 0.5 min = ~11.4 Wh) is 3-fold stronger than with the granulometer de-agglomeration system (75 W, 3 min = 3.75 Wh).

### 2.6. Particle Shapes

The QicPic granulomorphometer (Sympatec, Germany) with the MIWCELL cell dispersion module (wet analysis configuration) was used to measure the particle shapes by dynamic image analysis. The device is equipped with various lenses, and a digital camera captures the particles within the field of the lens. The lens needs to be selected to make the smallest particles visible and to find the right depth of field to focus on both larger and smaller particles. However, for widely dispersed samples, it can sometimes prove impossible to evidence all particles with the same lens. Here, the lenses M5 (measuring range: 1.8–3755 µm) and M3 (range: 0.55–1126 µm) were used. Samples were dispersed in 90% (*v*/*v*) ethanol added with 10% of methanol. The number of particles analyzed was between 1.10^5^ and 1.10^6^ for each sample. From the recorded images, we determined the median equivalent particle size and the aspect ratio (Equation (3))

(3) $$AR=\;\frac{Feret_{min}}{Feret_{max}},$$

where *Feret_min_* and *Feret_max_* correspond to the minimum and maximum distances between the two parallels restricting the image of the particle perpendicular to that direction. All measurements were made in triplicate

## 3. Results and Discussion 

### 3.1. Media Milling Motion and Energy Dissipated in Each Configuration

The collision points of the mobile media (ball, beads) in the system were extracted from DEM simulation to provide information on both media motion and energy dissipation during milling. In a real system, the contact points and transfers of energy depend on the material to mill, the milling media (ball, beads), the boundary conditions (jar), and the mechanical loading (vibration). Milling behavior is also strongly affected by filling ratio, PSD, temperature, relative humidity, and other factors. 

We can get a first evaluation of the level of energy dissipation for the single-ball configuration using an analytical solution, but it is much more complex for the multi-bead milling configuration. In a very simplified way, the energy transmitted into the system can be varied by the coefficient of friction and the coefficient of restitution (CoR) which controls energy dissipation during the collisions. As a first approach, energy dissipation was studied for CoR values varied from 0.5 to 0.9, corresponding to the extreme cases of wood–wood (close to the input raw materials to be milled) or steel–steel contacts, respectively. In the model, dissipation occurs during the contacts either through impacts or friction. For each contact k, the corresponding impact energy ($\varepsilon_{\mathrm{impact}}$) and frictional energy ($\varepsilon_{\mathrm{frictional}}$) can be calculated during contact time $t^{k}$ from the work of the normal $f_{n}^{k}$ and tangential $f_{t}^{k}$ components of the contact force and the normal $\delta_{n}^{k}$ and tangential $\delta_{t}^{k}$ elastic displacement during the contacts. Summing these work values for all contacts occurring during the simulation time $t_{s}$ gives:

(4) $$\varepsilon_{\mathrm{impact}}\left(t_{s}\right)=\underset{k}{\sum }\int_{0}^{t^{k}}f_{n}^{k}\delta_{n}^{k}\mathrm{dt},$$

(5) $$\varepsilon_{\mathrm{frictional}}\left(t_{s}\right)=\underset{k}{\sum }\int_{0}^{t^{k}}f_{t}^{k}\delta_{t}^{k}\mathrm{dt},$$

**Figure 2:**
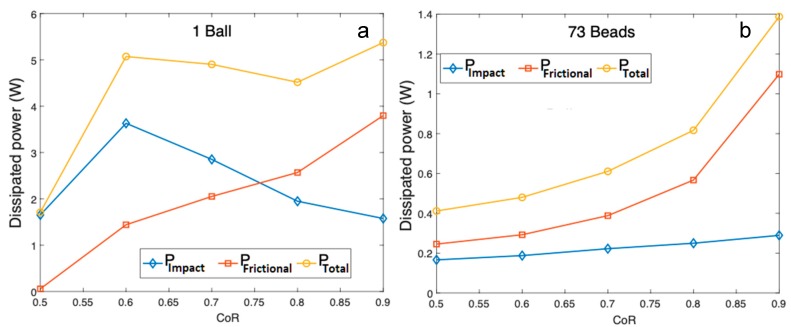
Dissipated power as a function of the coefficient of restitution (CoR) in (**a**) the single-ball configuration and (**b**) the multi-bead configuration.

These energies increase quasilinearly with $t_{s}$, showing that the dissipated power (which is the derivative of the energy as a function of time) remains constant during vibration of the milling jar. Figure 2 summarizes the dissipated power for each milling configuration and for CoR values in the range 0.5 to 0.9. 

In the multi-bead configuration, the energy dissipated by friction is greater than the energy dissipated by impact whatever the CoR. In the single-ball milling configuration, energy dissipated by impact dominated when the CoR was less than 0.75, but energy dissipated by friction dominated once CoR was more than 0.75. Indeed, for weak dissipation (i.e., an elastic material), the bulk of the impact energy is conserved, thus increasing the occurrence of collisions and increasing the dissipation by friction. Note too that total dissipated power was higher in the single-ball configuration.

**Figure 3:**
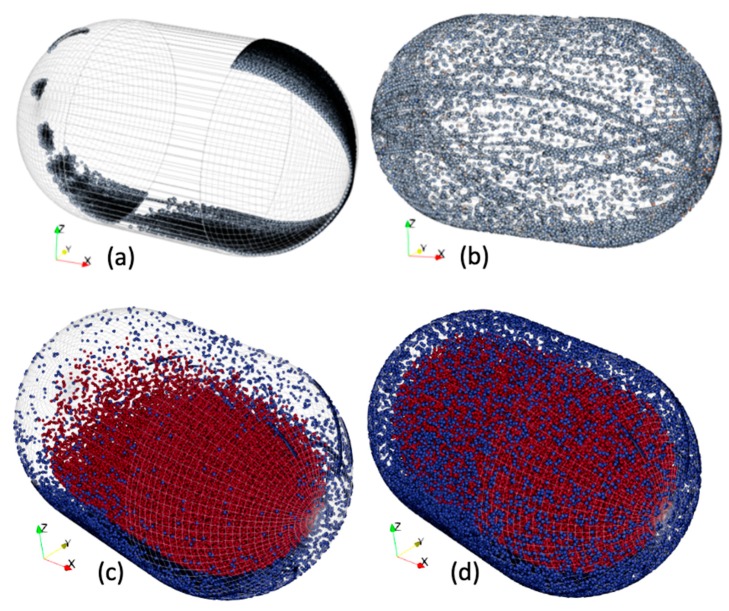
Monitoring of collisions during 30s: (**a**) single-ball configuration, CoR = 0.5; (**b**) single-ball configuration, CoR = 0.9, (**c**) multi-bead configuration, CoR = 0.5, (**d**) multi-bead configuration, CoR = 0.9.

Figure 3 shows the collision points for each milling configuration for a milling time of 30 seconds with CoR values of 0.5 and 0.9. In the multi-bead configuration (Figure 3c,d), red points are bead–bead collisions and blue points are bead–jar collisions. 

Note that CoR has a strong influence on the resulting collisions. The highest CoR leads to homogeneously-distributed collision positions. In the single-ball configuration, there was a ‘rolling’ behavior evidenced by continuous lines at the jar surface. Collisions were localized more to each end of the jar, although there were also impacts with the cylindrical part of the milling jar. In simulation, 54% of the impacts were localized on the spherical parts and the rest on the cylindrical part, and 62% of energy was dissipated by impact. The bulk of collisions occurred in the spherical parts (74%) of the jar.

With 73 beads, the milling mechanism seems rather different. A significant share of the collision points (62%) was localized at the center of the jar corresponding to bead–bead contacts. In this multi-bead configuration, collisions between the beads represent an average of 57% of total dissipated energy, of which 62% was dissipated by friction during bead–bead contact and the remainder by impact. In addition, bead–jar collisions represent 43% of total dissipated energy, of which 75% was dissipated by friction. In summary, 67% of the dissipation was by friction.

For a CoR less than 0.5, the ball tended to roll (Figure 3a). In the multi-bead configuration, the bead–jar collisions were mainly localized at the bottom of the jar. The beads that appeared to be trapped at the bottom of the jar had fewer and weaker impacts and energy was mainly dissipated by friction. 

In the experimental tests, the fact that milling conditions (filling ratio, particle size, density of the powder bed) evolved implies that the collisions and the proportions of the energy dissipated by impact ($\varepsilon_{\mathrm{impact}}$) and by friction ($\varepsilon_{frictional}$) also evolved. However, the presence of powder inside the jar had a substantial damping effect, and the CoR was probably closer to 0.5 (cushioned impact) than 0.9 (elastic impact). Based on this assumption and on the simulation results, we assume in the following that the energy is mainly transmitted to the powder by impact in the single-ball configuration and by friction in the multi-bead configuration.

### 3.2. Particle Size Distributions

**Figure 4:**
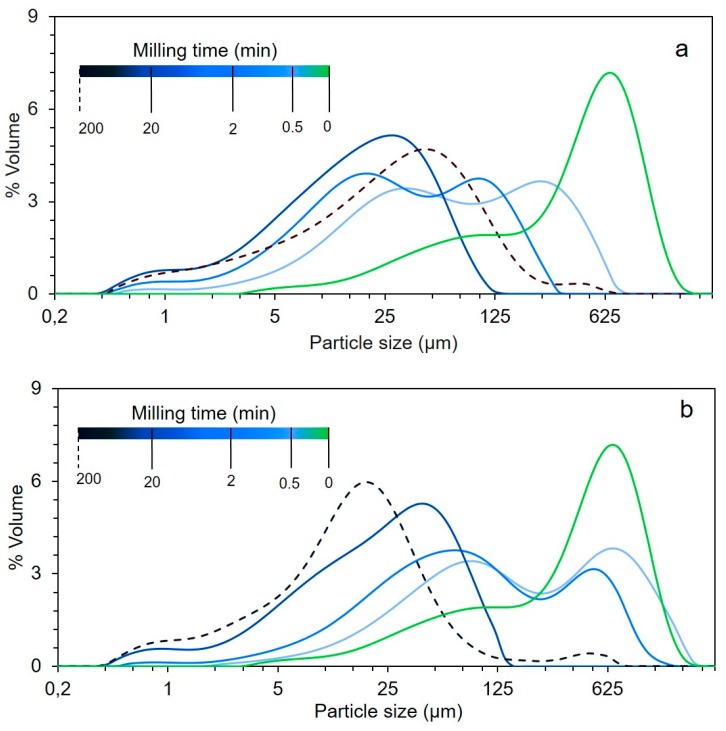
Evolution of the particle size distribution as a function of grinding time in a single-ball (**a**) and multi-beads configuration (**b**).

Figure 4a,b show the evolution of PSD in powders milled by a single-ball and by 73 beads. Each curve corresponds to a different milling time—darker curves correspond to longer milling times. 

In the single-ball configuration where milling energy is mostly transmitted by impact, milling first produced two populations of equivalent volume (centered around 275 µm and 30 µm, respectively). At longer milling times, these two populations shifted to the finest particle range, driven by the production of an increasing quantity of fine ~1 µm-diameter particles. As PSD are expressed in volume, the ~1 µm peak corresponds to a significant number of very fine particles. These evolutions can be related to an almost instantaneous division of particles under the action of vigorous impacts. 

In the case of multi-bead milling, the peak centered at 700 µm, corresponding to the main particle population in the initial powder, gradually disappeared to first produce an increasing quantity of particles at 100 µm. At longer milling times, we observed a similar phenomenon, with the population of ~100 µm particles disappearing into a population of finer ones (~5 µm and 1 µm). This could be explained by a significant share of friction energy in total milling energy in this multi-bead configuration, as calculated from the numerical model (Figure 2a,b). The initial particles were likely eroded by friction during the bead–bead and/or bead–jar collisions. Comminution by friction could thus be assimilated to an attrition mechanism. 

The differences in PSD between the two configurations gradually diminished over milling times. At 200 min of milling (the dotted-line curve), the PSD was seen to shift towards coarse particles. In the single-ball configuration, this consisted of a shift of mean peak from 30 µm (20 min of milling) to 45 µm (200 min of milling) and the emergence of a new population at around 480 µm. In the multi-bead configuration, the size of the main powder population continued to decrease, but a new larger population also emerged. The creation of coarser particles from smaller ones has already been reported with ultrafine milling of mineral materials [17] and lignocellulosic materials such as wood powder [18,30,31]. This was interpreted as the formation of agglomerates of fine particles produced during the milling process. 

### 3.3. Evolution of Particle Shapes during Milling

**Figure 5:**
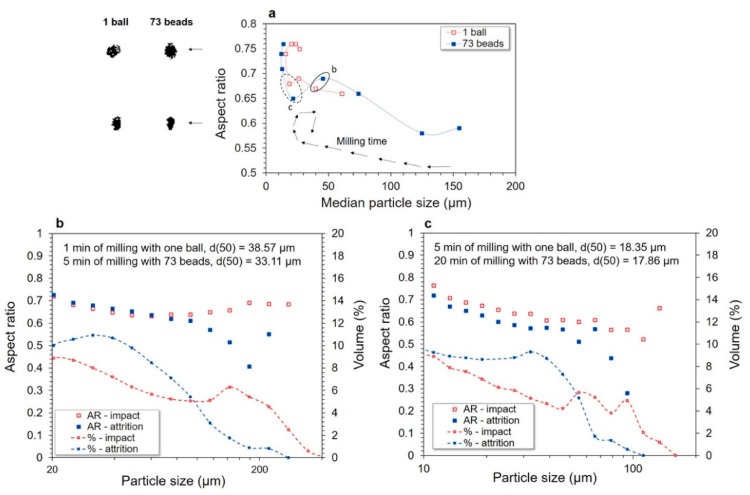
Evolution of aspect ratio (AR) as a function of median particle size (**a**). Percentage of each particle size population in the two configurations for different milling times: 1 min in single-ball configuration and 5 min in multi-bead configuration (d50 ≈ 35 μm) (**b**); 5 min in single-ball configuration and 20 min in multi-bead configuration (d50 ≈ 18 μm) (**c**).

The evolution of particle shape during milling was monitored via aspect ratio (AR). An elongated or needle-like particle will have an AR closer to 0, whereas a perfectly spherical or cubic particle will have an AR equal to 1. As the comminution-process kinetics are different in the two configurations, Figure 5a plots the median aspect ratio as a function of median particle size. The black arrow indicates the course of the comminution process and evidences the occurrence of agglomeration phenomena when a new increase in median particle size is observed. In order to display the shapes associated with the different AR, illustrative examples of particles extracted from the analysis are pictured on the left of Figure 5a for each configuration. 

Mean AR increased from 0.60 to 0.78 during the whole milling process as median particle size decreased. This AR increase remained very weak for particles with median sizes above 20 μm but became clear-cut when the agglomeration phenomena appeared (particles with a median size below 20 μm), suggesting that agglomerates tend to be more spherical than particles from initial fragmentation. Interestingly, for a same median particle size, the median AR was similar whatever the milling configuration. 

However, AR at median particle size is not representative of the diversity of particle shapes that can coexist in the powder. In Section 3.2, PSD analysis evidenced the coexistence of several populations whose proportions differed according to milling configuration. To highlight potential differences in particle morphologies between these populations, Figure 5b,c plot the particle size and AR distributions obtained in each configuration for the same median particle sizes (35 µm and 18 µm). As the kinetics of milling is slower in the multi-bead configuration, the milling time to reach the target particle size is not the same in the single-ball configuration as in the multi-bead configuration, i.e., 1 min and 5 min to reach 35 µm in Figure 5b and 5 min and 20 min to reach 18 µm in Figure 5c, respectively. The *x*-axis scale was adapted to the detection range of the lens used in each case (i.e., from 10 μm to 625 μm in Figure 5b and from 20 μm to 625 μm in Figure 5c). Note the difference in aspect of the PSDs obtained with the QicPic analyzer vs. the laser granulometer, which is related to the difference in size classes for the two measurements and to the semi-log plot with different data ranges. Interestingly, the AR of the smallest particles were always very similar whatever the configuration and milling time considered (particles below 100 μm in Figure 5b and below 40 μm in Figure 5c), which highlights that for the smallest particles, particle shape is not a discriminant factor between the single-ball and multi-bead configurations.

In Figure 5b, the AR in the single-ball configuration appears quasi-similar for all particles (around 0.7) whatever their size, suggesting that the loading mode generated by the motion of a single ball “bursts” the raw particles into smaller particles that still share similar shapes. Conversely, in the multi-bead configuration, the AR of the particles was lower and globally increased from 0.5 to 0.7 (at 20 µm), although some variations were observed. It can be assumed in this case that the motion of the beads gradually eroded the raw particles (that get out of the QicPic device detection range) and tore them into smaller ones with various shapes depending on their sizes. Figure 5c also shows this pattern at 5 min in single-ball configuration and at 20 min in multi-bead configuration for a median particle size of approximately 18 µm. However, the AR of the smallest particles (10 μm) was around 0.8, which points to rounding-off effects as particle size decreased [32,33] that could also enhance agglomeration phenomena. 

### 3.4. Evolution of Specific Surface Areas (SSA) and Particle Agglomeration

SSA (developed surface area per mass of sample), which is related to the PSD of the powder, integrates all the distribution characteristics into a single value. For a given powder mass, a larger SSA reflects a greater proportion of fine particles. 

**Figure 6:**
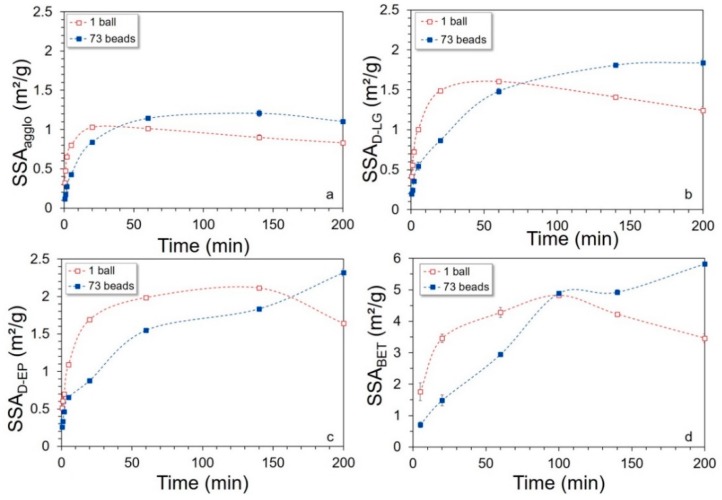
Evolution of the different specific surface area (SSA) as a function of milling time in single-ball and multi-beads process configurations: without de-agglomeration (**a**); after de-agglomeration using the granulometer system (**b**); after de-agglomeration using the external probe (**c**); with the Brunauer–Emmett–Teller (BET) gas-sorption method (**d**).

The time–course evolution of powder SSA through milling in both configurations was measured via various techniques and plotted in Figure 6. Note that Figure 6 does feature some error bars, but they are very small (i.e., less than 5%) and not always visible on Figure 6a–c. 

A first set of data compared the SSA obtained from the laser granulometer (an integration of the surface of all detected particles, assuming spherical shapes) after different intensities of mechanical de-agglomeration: SSA_agglo_ (*no de-agglomeration* step, Figure 6a), SSA_D-LG_ (*ultrasonic de-agglomeration* using the integrated *granulometer system*, Figure 6b), SSA_D-EP_ (*ultrasonic de-agglomeration* using an *external probe*, Figure 6c). We also performed another type of SSA measurement, i.e., SSA_BET_ (Figure 6d), that does not sum each particle surface but directly integrates the total surface area of the powder available to gas sorption. 

The direct measurement of SSA from the laser granulometer (Figure 6a) showed a rapid increase in SSA_agglo_ during the first few minutes of milling followed by a slowdown then a decrease at longer milling times. As observed in the particle size evolution, the single-ball configuration led to a surface development that was initially faster but then became limited earlier than with a multi-bead set-up. A similar SSA_agglo_ of approximately 1 m^2^·g^−1^ was reached after 15 min with a single-ball mill but required 40 min of milling with a multi-bead mill, as SSA_agglo_ increase stopped later on, after about 60 min of milling, and then remained stable. After 200 min of milling, the multi-bead configuration overlapped with the single-ball SSA_agglo_ performance by up to 20%. This appears to confirm our previous hypotheses on mechanical stress effects, with a single ball proving initially more efficient, probably due to its more energy-effective impacts, whereas the multiple beads showed a later efficiency of multiple but weaker impacts and frictions from the beads to wear down smaller particles.

Granulometry results after a moderate preliminary ultrasound treatment of the powders (Figure 6b) showed similar trends in milling kinetics, but with SSA_D-LG_ reaching higher levels (~1.5 m^2^·g^−1^ vs. ~1 m^2^·g^−1^ maximum SSA_agglo_ in the single-ball configuration). In this case, the decrease in SSA_D-LG_ through ongoing kinetics occurred later on, i.e., after 60 min with a single ball. Interestingly, in the multi-bead configuration, SSA_D-LG_ increased continuously up to the end of the experiment even though it decelerated after 60 min. Increasing the ultrasound power when using the external probe (Figure 6c) led to even larger SSA (>~2 m^2^·g^−1^ maximum in the single-ball configuration), but the kinetics in this case followed a different pattern. Here, the SSA_D-EP_ from single-ball experiments increased up to 140 min then decreased only in the last sample at 200 min. In the multi-bead configuration, the SSA_D-EP_ increased continuously throughout milling. At the end (200 min), ~30% more particle surface was obtained with the multi-bead configuration than with a single ball.

The differences in SSA between these mechanically de-agglomerated samples show that (i) there is competition between fragmentation (SSA increase) and agglomeration (SSA decrease) throughout milling of this biomass sample in a vibratory ball mill, (ii) the agglomeration occurs differently in different milling configurations, with a single ball (predominance of impact) being more conducive to agglomeration than multiple beads (predominance of attrition), (iii) applying differential ultrasound power can reveal agglomerates of different strengths.

As shown in Figure 6d, the SSA calculated based on BET gas-sorption technique give much higher values than reported above, with e.g., ~5 m^2^·g^−1^ maximum SSA_BET_ for single-ball milling, which is more than twice as high as the SSA_D-EP_. This gap can be explained by the differences in principles of the methods: the BET technique accounts for the entire surface of all particles, including their accessible pores, whereas with laser granulometry (i) only the particles falling into the detection range of the apparatus are considered, thus excluding the finest-ground particles, and (ii) the SSA is calculated based on the assumption of sphere-shaped particles as default option. However, the time–course pattern of SSA through milling is very similar between BET and external ultrasonic probe-assisted measurements. This indicates that gas can access particle surfaces even in partially-agglomerated powders. In the single-ball configuration, SSA_BET_ decreased after 100 min of milling. This means that less gas was able to penetrate the powder matter, which could reflect a change in the nature of agglomerates beyond this threshold. In the multi-bead configuration, SSA_BET_ increased almost linearly with milling time, evidencing a difference in nature of agglomeration in the powders according to milling configurations. Opoczky [8] reported similar observations concerning silicate milling and distinguished agglomerates from aggregates depending on their degree of cohesiveness. Similarly, Nichols et al. [7] proposed to keep the term ‘agglomerates’ strictly for particle clusters but to distinguish soft agglomeration from hard agglomeration depending on cluster cohesive strength. 

**Figure 7:**
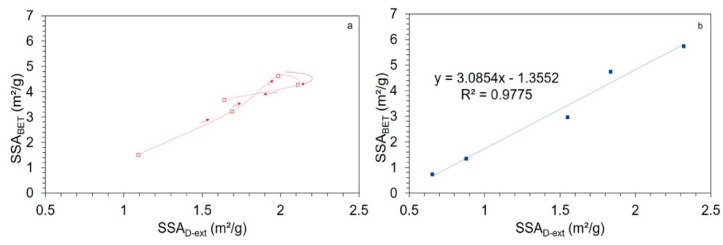
Correlations between SSA obtained by the ultrasound external probe technique and SSA obtained by the BET technique on powders resulting from single-ball (**a**) and multi-bead (**b**) milling configurations. Arrows indicate time–course advancement in the milling process.

Figure 7 plots the correlation curves between SSA_D-EP_ and SSA_BET_ for both loading configurations.

In the multi-bead configuration (Figure 7b), we found a linear correlation for all points along the milling experiment. This result show that the mechanical energy delivered by the external ultrasonic probe does not break down native bark material in the applied conditions and is only efficient for dispersing secondary particle clusters, since even at long milling times, SSA_D-EP_ did not follow a different trend to SSA_BET_. In the single-ball configuration (Figure 7a), we again found a linear correlation, but only in the so-called ‘soft-agglomeration’ domain, after which the values from the two methods diverged when the previously-defined ‘hard-agglomeration’ domain was reached. Therefore, the external ultrasonic probe procedure appears to be an efficient and simple method to gauge the total level of agglomeration within this kind of powder, and furthermore it can be combined with gas-sorption methods to distinguish between agglomerates of different mechanical strengths which likely result from different physical and chemical clustering mechanisms. Concerning the two milling configurations used here, although both produced considerable particle agglomeration, it is apparent that the impact-dominant mode (single-ball configuration) results in agglomerates of different types (at least two, soft and hard) with increasing hardness as milling proceeds, whereas only weaker soft-agglomerates are found with the attrition-dominant (multi-bead) mode.

### 3.5. Successive Mechanical Stresses

Here, in the context of milling lignocellulosic biomass, we found that the single-ball configuration (impact-dominant mode) can first rapidly reduce the particle size of the starting material, after which the multi-bead configuration (attrition-dominant mode) could efficiently complete the comminution process while limiting agglomeration phenomena. To validate this hypothesis, were tested single-ball milling followed by multi-bead milling as well as the reverse combination (multi-bead milling followed by single-ball milling). The experiments were conducted on the basis of a 200 min total milling time: (1) first 60 min in single-ball configuration (corresponding to the onset of significant agglomeration) then 140 min in multi-bead configuration (‘ball + beads’); (2) first 65 min in multi-bead configuration (same SSA as with 60 min with a single ball) then 135 min in single-ball configuration (‘beads + ball’). The reference samples used to estimate the effects of these mechanical-stress combinations were 200 min milling experiments with only a single-ball and with only a multi-bead configuration.

**Figure 8:**
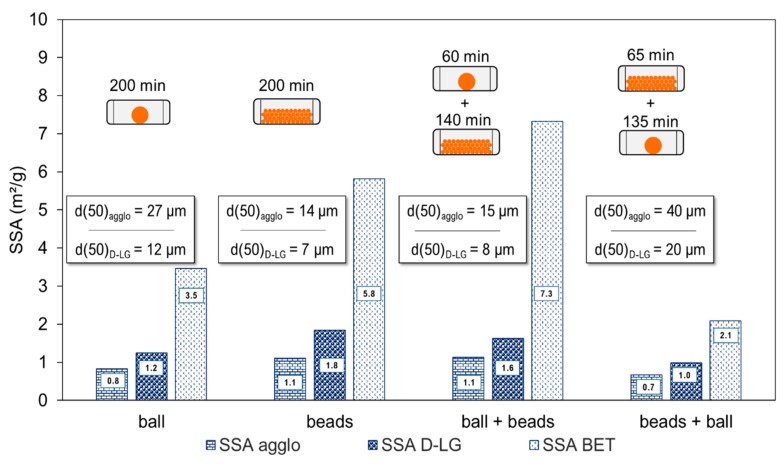
SSAs of powders obtained by single and successive mechanical stresses.

Figure 8 charts the SSA obtained after 200 min without de-agglomeration (Agglo), after de-agglomeration with a granulometer (D-LG), and by the gas-sorption technique (BET), together with the median particle sizes for each milling configuration.

Simple attrition (multi-bead) and *impact + attrition* (ball + beads) millings afforded the smallest (and similar) d50 in agglomerated (14 µm, 15 µm) or de-agglomerated form (7 µm, 8 µm). The *impact* (ball) and *attrition + impact* powders (beads + ball) had a significantly higher d50. 

The SSA_BET_ of the *impact + attrition powder* (ball + beads) was by far the largest of all the samples (7.3 m^2^·g^−1^), followed by *attrition powder* (multi-bead) (5.8 m^2^·g^−1^), then *impact powder* (single-ball) (3.5 m^2^·g^−1^), and lastly *attrition + impact powder* (beads + ball) (2.1 m^2^·g^−1^). As seen earlier, an *impact powder* may contain strongly cohesive clusters, called hard agglomerates. Here, the *attrition + impact powder* gave an even lower SSA_BET_ than the corresponding *impact powder*. This indicates that this loading combination promotes a strong hard agglomeration at long milling times.

Conversely, the median particle sizes of the *impact + attrition powder* highlight the propensity of attrition to generate finer particles while limiting agglomeration. Note too that SSA_D-LG_ was significantly smaller for *impact + attrition powder* than for only *attrition powder* (1.6 vs. 1.8 m^2^·g^−1^). This indicates that more agglomerates in the *impact + attrition powder* resisted the weaker mechanical de-agglomeration of the granulometer device than in the *attrition powder*, but still with a soft agglomerate typology as they allow gas penetration in the BET measurement.

Thus, the degree of agglomeration in the powders is dependent on the loading stress and the sequence of the operations. Essentially, attrition tends to generate large amounts of fine particles whereas impact tends to promote their agglomeration. This makes it important to optimally sequence of loading modes according to the process objectives, i.e., fast reduction, fines production, control of agglomeration, etc.

Table 1 compares the efficiency of the different loading modes throughout milling by calculating the reactive surface (i.e., surface accessible to the gas in the BET technique) that could be generated with 1 kWh of milling energy. 

For each configuration (single-ball or multi-bead), the efficiencies decreased steadily from beginning to end, due to the high energy cost of breaking down the smallest particles. *Impact* mode appears to be more energy-effective than *attrition* in the first stage of the milling process. This trend reversed after 60 min and was amplified strongly up to 200 min. In this context, the combination of *impact + attrition* proved the most efficient process at 200 min of milling, demonstrating good complementarity between these successive stresses. The other combination (*attrition + impact*) was less effective than *attrition* alone due to the impact-driven agglomeration, but was still better than *impact* alone due to the large amount of fines generated by the first *attrition* step.

These results consolidate our earlier observations on the differences between the two loading modes and their possible complementarity. On the one hand, attrition is under-efficient on the fragmentation of coarse raw particles but effective for quickly producing fine particles and limiting agglomeration phenomena. On the other hand, an impact can quickly and effectively reduce the initial particle sizes, but it loses efficiency as milling proceeds. In our study, a SSA_agglo_ threshold of ~1.03 m^2^·g^−1^ marks the limit when the agglomeration process becomes predominant over fragmentation in an impact-loading mode. At this point, the more efficient option to complete exhaustive milling is to switch to an attrition-loading mode. 

## 4. Conclusions

Here we evidenced differences in biomass powder properties and in milling energy consumption depending on loading-mode configuration. A single-ball configuration (impact mode) is more effective for fragmenting coarse particles but less efficient for comminution of fine particles due to agglomeration phenomena. Conversely, attrition of a multi-bead configuration (attrition mode) appears to be more efficient to reduce the size of fine particles and produces low-agglomerative powders. However, these limitations can be overcome by combining successive mechanical stresses. First, impact mode enables a rapid reduction of particle size, then attrition mode enables further size reduction of the finer particles while avoiding hard agglomeration.

## Figures and Tables

**Table 1:** Surface energy of the different configurations.

Configuration	Reactive Surface Per kWh (m^2^_BET/_ kWh)		
	0→60 min	0→120 min	0→200 min
Ball	133	75	34
Beads	112	88	68
⅓ of time ball + ⅔ of time beads			82
⅓ of time beads + ⅔ of time ball			59
